# Nest Bacterial Environment Affects Microbiome of Hoopoe Eggshells, but Not That of the Uropygial Secretion

**DOI:** 10.1371/journal.pone.0158158

**Published:** 2016-07-13

**Authors:** Ángela Martínez-García, Manuel Martín-Vivaldi, Sonia M. Rodríguez-Ruano, Juan Manuel Peralta-Sánchez, Eva Valdivia, Juan J. Soler

**Affiliations:** 1 Estación Experimental de Zonas Áridas (CSIC) E-04120 Almería, Spain; 2 Departamento de Zoología Universidad de Granada, E-18071 Granada, Spain; 3 Departamento de Microbiología Universidad de Granada, E-18071 Granada, Spain; National Research Laboratory of Defense Proteins, REPUBLIC OF KOREA

## Abstract

The study of associations between symbiotic bacterial communities of hosts and those of surrounding environments would help to understand how bacterial assemblages are acquired, and how they are transmitted from one to another location (i.e. symbiotic bacteria acquisition by hosts). Hoopoes (*Upupa epops*) smear their eggshells with uropygial secretion (oily secretion produced in their uropygial gland) that harbors antibiotic producing bacteria. Trying to elucidate a possible role of nest material and cloaca microbiota in determining the bacterial community of the uropygial gland and the eggshells of hoopoes, we characterized bacterial communities of nest material, cloaca, uropygial gland and eggshells by the ARISA fingerprinting. Further, by adding material with scarce bacteria and antimicrobial properties, we manipulated the bacterial community of nest material and thus tested experimentally its effects on the microbiomes of the uropygial secretion and of the eggshells. The experiment did not influence the microbiome of the uropygial secretion of females, but affected the community established on eggshells. This is the first experimental evidence indicating that nest material influences the bacterial community of the eggshells and, therefore, probability of embryo infection. Some of the bacterial strains detected in the secretion were also in the bacterial communities of the nest material and of cloaca, but their occurrence within nests was not associated, which suggests that bacterial environments of nest material and cloaca are not sources of symbiotic bacteria for the gland. These results do not support a role of nest environments of hoopoes as reservoirs of symbiotic bacteria. We discuss possible scenarios explaining bacterial acquisition by hoopoes that should be further explored.

## Introduction

Exploring the influence of bacteria on animal health and evolution is nowadays of central importance for life sciences [1]. The most known impacts of bacteria are the detrimental effects of some strains, but animals may also benefit from others. Most bacteria produce defensive compounds that inhibit antagonistic competing microorganisms [2], including host pathogens [3]. Apart from the effects of individual bacterial strains, the diversity and structure of the complete bacterial communities may also have important implications for health of hosts [4].

Detecting associations between symbiotic bacterial communities of hosts and those of surrounding environments is particularly exciting because it would help to understand how bacterial assemblages are acquired and how they are transmitted from one location to another (i.e. bacteria acquisition) [5]. The same bacterial strains may have beneficial or detrimental effects for hosts depending on the bacterial community (i.e., location) to which they are incorporated. Pathogenic bacteria may for instance have no detrimental effects when included within the gut microbiota of animals, but on eggshells would increase probability of embryo infection [6,7]. Moreover, bacteria with negative effects such as feather degraders in adult’s primary feathers [8] have been demonstrated to benefit hosts when incorporated to the nest or eggshell bacterial communities by using nest lining feathers [3,9,10]. Thus, exploring host colonization by microorganisms would contribute to the understanding of ecological processes for pathogenic contamination of hosts, mechanisms of mutualistic-bacteria acquisition by host, and their effects on host bacterial communities [1].

The avian nest includes several environments (i.e., the nest cup with their building materials [11], the eggshells, the skin of adults and nestlings, or the cloaca and feces [12]), which are likely associated with particular bacterial communities that interact to each other [5]. The strength of the interactions would depend on particularities of bacterial communities and on factors affecting transmission among locations [5]. Climatic conditions (i.e., temperature and humidity [13,14]), life history characteristics [15], some behavioral defensive traits (i.e. incubation [16–18], or the use of material with antimicrobials [10,19,20]) are also known as determinants of nest bacterial communities and, therefore, would affect relationships among nest microbiomes. As far as we know, the bacterial assemblages present on the different nest components have only been explored within nests of reed warblers (*Acrocephalus scirpaceus*) [5]. The study showed that some bacteria are transmitted from nest material to the eggshell, and that incubation affects the microbiome on the eggshell (see also [17,18]). However, in these studies, the importance of the eggshell microbiomes is only considered for its possible pathogenic effects over embryos. The uropygial secretion of some avian species includes microbes [21–23] that would interact with nest microbiomes. In the case hoopoes (*Upupa epops*), we know that females paint their eggs with uropygial secretion containing those symbiotic bacteria [24,25] that influence bacterial community of the eggshell [26] and probability of trans-shell embryo infection [25]. Thus, bacterial communities of the uropygial secretions should also be considered when exploring factors affecting bacterial assemblage at different nest environments.

We here explore the associations between bacterial communities of different environments of hoopoe nests. Hoopoes do not build nests, but use cavities that frequently had been used by conspecifics or other bird species for breeding. Thus, nest materials from old nests or remains (feathers, feces, etc.) from previous reproduction events (hereafter, nest materials) may be a source of microorganisms for bacterial communities of new active nests. We already know that some of the bacteria of the uropygial secretion are transmitted to the eggshell [26]. However, the contribution of nest materials and cloaca as sources for the bacterial communities of the eggshells and uropygial secretion of hoopoes is still unknown. Here, by adding nest materials with reduced bacterial loads, we manipulated bacterial communities of nest material in a group of nests and characterized the bacterial communities of different nest environments (i.e., nest material, cloaca, uropygial secretion and eggshell) of control and experimental nests of hoopoes (*Upupa epops*).

Our aims here are two folds. First we test the hypothetical role of nest material and of cloaca fluids as sources of symbiotic bacteria for the uropygial gland of females and nestling hoopoes. This scenario therefore predicts positive associations between the bacterial community of the uropygial secretion and those of nest materials and cloaca. The second aim is to investigate the hypothesis that nests remains and/or cloaca microbiota contribute to the eggshell bacterial community of hoopoes. Thus, we predicted that the experimental modification of nest material should influence eggshell bacterial communities, and positive associations between bacterial communities (i.e. prevalence of bacterial strains) of nest material and cloaca with that of the eggshell.

## Materials and Methods

### Ethics statement

The study was performed according to relevant Spanish National and regional guidelines (Decreto 139/2010 de 13 de Abril). The protocol was approved by ethics committee of Spanish National Research Council (CSIC) and all necessary permits to perform this research were provided by the Consejería de Medio Ambiente of the Junta de Andalucía Spain (Ref: SGYB/FOA/AFR). The study was approved by the Ethics Committee of the University of Granada (Comité de Ética en Experimentación Animal, CEEA, Ref.: 785).

The time spent in each hoopoe nest was the minimum necessary for bacterial sampling and for treatment application. This experiment did not show detectable effects in adult incubation behaviour or egg viability.

### Study species and study area

The hoopoe is distributed throughout Europe, Asia and Africa, inhabiting open woods or open areas as steppes, grasslands, pastures, semi-deserts, or crops whenever they have scattered trees, walls or buildings providing holes for nesting and soil without tall vegetation for feeding [27–29]. The uropygial secretion of hoopoe females but not that of males experiences apparent seasonal changes [30]. Uropygial secretion of nesting females and nestlings are malodourous, of brown-greener coloration, and contains a large amount of bacterial symbionts that produce antimicrobial substances [21–23,30–32]. Female hoopoes besmear the eggshells with the antimicrobial secretion which accumulates in special crypts [25], turning the egg color from pale-blue to brown greenish [24] and protecting the embryo from trans-shell pathogenic infection [25,31]. Females lay one or two clutches of 6–8 eggs along the breeding season, between February and July [33]. Incubation lasts 17 days and starts with the first or second egg, which results in eggs hatching asynchronously at 24 h or even greater intervals [34].

The study was performed in 2011 in a population maintained in captivity since 2008. The captive pairs were distributed in two localities with appropriate facilities; one in the Hoya of Guadix (37°18′N, 38°11′W, Granada province, southern Spain) and the other one in Almería (36°50´N, 2°28´W, Finca Experimental La Hoya, EEZA-CSIC). All females were ringed with numbered aluminum and plastic color rings for individual recognition. Breeding pairs were housed in independent cages at least 3m x 2m x 2m installed in the open, scattered and isolated to avoid interactions between pairs and ensure successful breeding. Cages had access to soil and provided with live food (crickets, vitamin-enriched fly larvae and meat (beef heart)) *ad libitum*, and were visited daily from mid-February to the end of July.

### Experimental design and sampling

Before reproduction started, we collected nest material from 15 nest-boxes that hoopoes used for reproduction during 2010 in the Guadix wild population. The material from each nest-box was individually stored in labeled bags at room temperature until their use in our captive population. Experimental breeding pairs were randomly assigned to control and experimental treatments. The nest-boxes installed in the cages for this study had never been used by any bird species for breeding. Each control pair received a nest-box filled with a 3 cm layer of material collected from one nest box of our wild population. Experimental pairs on the contrary received a nest-box filled with a similar amount of commercial crushed and mashed olive (*Olea europaea*) stones (used as combustible for heating in Spain). This kind of material is not expected to harbor the typical bacterial community living within hoopoe nests. Moreover, it is known that olives contain substances (i.e. oleuropein) with high antimicrobial activity [35–37].

We found different bacterial load among control and experimental nest materials used in the experiment (S1 Appendix). To test for the antimicrobial properties of the experimental nest material, we performed antagonistic tests with crushed olive stones against several indicator bacteria. We found high grow inhibition capacity of this material for most tested bacterial strains (Fig A in S2 Appendix). All these results confirm the assumption that experimental and control nests greatly differ in their initial microbial community.

Control and experimental nest-boxes were fastened to cages one day before the experimental hoopoe pairs were released inside. The experiment involved 30 nest boxes (15 for each treatment), but for six of them the genetic analyses failed for at least one of the considered communities (nest material, secretion, cloaca and/or eggshells). Since we were interested in within nest associations of bacterial communities, for statistical analyses we only considered the 24 nests (12 of each experimental treatment) with complete information.

Nest material was sampled the same day that it was introduced in nest-boxes (day 0). We collected samples of nest materials by hand with sterile latex gloves and stored them in Falcon tubes with 15 ml of sterile sodium-disodium phosphate buffer (Na_2_HPO_4_ 0.1 M and NaH_2_PO_4_ 0.1 M, pH 7.1). Bacterial communities of the uropygial gland, the cloaca of females, and the eggshells were sampled 14 days after the first egg was laid. Incubating females were caught by hand and after sampling bacterial communities, were released again within the nest box to reduce disturbance. We wore new sterile latex gloves cleaned with 70% ethanol for the whole process of sampling, in order to avoid bacterial contamination among nests.

Before sampling the uropygial gland (with alert animals), the circlet and surrounding skin of the uropygial gland were softly washed with a cotton swab soaked in 70% ethanol to reduce the risk of contamination of the secretion with external bacteria. After evaporation of the ethanol, a sterile micropipette tip (1–10 μl micropipette, Finpipette) was introduced in the gland papilla after opening the circlet of feathers that cover the gland entrance. The papilla was pressed softly with a finger to collect the entire secretion available. The secretion was transferred to a sterile microfuge tube and, afterwards, 5 μl were separated in a different sterile microfuge tube for the analyses. The cloaca microbiota was sampled by introducing and repipetting three times 500 μl of sterile phosphate buffer in the cloaca. We used sterile tips and automatic pipettes (100–1000 μl micropipette Finpipette). Samples were stored in sterile microfuge tubes (for further information on this methodology see [38]). Bacterial samples of eggshells of the entire clutch were collected by completely cleaning the surfaces of all eggs with the same sterile swab slightly wet with sterile phosphate buffer. These samples were individually stored in sterile Eppendorf tubes within 1.2 ml of buffer solution (see [15]). All samples were kept cool (i.e. 1-3°C) until storing them in the lab at -20°C in the same day of sampling for molecular analyses.

### Laboratory work

We used different bacterial genomic DNA extraction protocols depending on the type of sample. DNA from nest material samples was extracted from 0.25g of homogenized nest material per sample using the PowerSoil^®^ DNA Isolation Kit (MO BIO Laboratories, Inc., Carlsbad, USA), following the manufacturer’s instructions. The viscous secretions of the uropygial gland and cloaca samples were extracted with FavorPrep™ Blood Genomic DNA Extraction Kit (Favorgen Biotech Co., Ping-Tung, Taiwan). Finally, eggshells DNA was extracted with a specific procedure for obtaining genetic material from swabs named Chelex-based DNA isolation [39].

Automated Ribosomal Intergenic Spacer Analysis (ARISA) [40] was used to characterize the composition of bacterial communities. ARISA amplifies an intergenic transcribed spacer (ITS) region between the prokaryotic 16S and 23S rDNA. This region is highly variable both in size and sequence between species, offering higher taxonomic resolution than other techniques [41]. The ITS was amplified using the primer pair ITSF (5´-GTCGTAACAAGGTAGCCGTA-3´) and ITSReub (5´-GCCAAGGCATCCACC-3´) [42]. The primer ITSReub was labelled fluorescently with 6-FAM. Amplifications were performed in 50 μl reaction volumes containing ultrapure H_2_O, 20 μl of 5 PRIME MasterMix (2.5x) including 1.5mM Mg(OAC)_2_, 200 μM dNTPs, 1.25 U Taq DNA polymerase, 0.2 μM of primers and 5μl of diluted DNA 1:10. PCRs were carried out in an Eppendorf Mastercycler Nexus Family. Fragments were amplified under the following conditions: initial denaturation at 94°C 2 min, followed by 30 cycles with denaturation at 94°C 45 s, annealing at 52°C 45 s and extension at 72°C 1 min, with a final extension at 72°C 5 min. Amplified PCR products were diluted 1:10 and denatured by heating in formamide. Fragment lengths were determined by mean of automated fluorescent capillary electrophoresis on a 3130 Genetic Analyzer (Applied Biosystems). Electropherogram peak values were calculated after interpolation with an internal size standard named GeneScan™ 1200 LIZ dye Size Standard (Applied Biosystems). These analyses were performed at the Scientific Instrumentation Center of the University of Granada.

### Statistical analysis

Peak Scanner v1.0 (Applied Biosystems) was used to determine fragment lengths identifying different bacterial Operational Taxonomic Units (hereafter, OTUs) within each sample. Scripts in R-environment [http://cran.r-project.org/]) available at http://www.ecology-research.com, were used for binning DNA fragment lengths from different samples. Binning exercise was performed by stablishing a window size of 4 base pairs (bp) and a distance of two consecutive binning frames (i.e. shift) of 0.1. We only considered peaks with values of relative intensity of fluorescence larger than 0.09% and fragments above a threshold of 50 fluorescence units that ranged between 100 and 1,000 bp [43]. We used the presence-absence matrix generated after binning process for the analyses. Molecular fingerprinting techniques are highly reproducible, robust, and have been proven useful for comparative analysis of microbial community structure [44,45].

Because of the possible differential experimental effects on microbial communities of each sampled site, they were described (i.e. richness, OTU’s prevalence, nestedness) and compared to each other including only control nests. We used General Linear Models (GLMs) to explore the effects of sampled sites (nest material, cloaca, uropygial gland and eggshells) and experimental treatments (experimental *vs* control) on bacterial richness (i.e. number of OTUs per sample) and nestedness.

We are particularly interested in testing whether nest materials or cloaca microbiotas may be sources of the symbiotic bacteria that install in the uropygial gland of hoopoes. Hence, for these analyses we only considered OTUs that were detected in the bacterial community of the uropygial secretion. In addition, to explore the within individual association in OTUs prevalence among different sampled sites, we built 2x2 contingence frequency tables for every OTU and pair of sampled sites. Nestedness estimations for each sampling event were explored by calculating the NODF index (nestedness based on overlap and decreasing fill) [46,47] with the web interface NeD (http://purl.oclc.org/ned) developed by [48]. We organized matrices for each sampling occasion (individual females during a single reproductive event) as including all bacterial strains (OTUs) detected in the sampled sites. Sampled sites of bacterial communities were in columns ordered following the expected direction of nestedness, and OTUs identities were therefore organized as rows [46,47,49]. To test for the possible origins of bacteria in the uropygial gland, we explored whether the bacterial community of the uropygial secretion was nested within those of nest materials, and/or cloaca.

We have previously shown that the bacterial community of the eggshells of hoopoes is nested within those of the brood patch and/or beak, and those are nested within the bacterial community of the uropygial secretion of hoopoes [26]. Here, to explore the possibility that the community on the eggshells also receive strains from nest materials and/or the cloaca, we tested for its nestedness within those sampled sites. Briefly, we estimated NODF for columns for each of the study nest, which inform of nestedness of communities among sampling places [46]. Z-values estimated of NODF indicate the existence of nested if value is higher than 1.64 (p < 0.05) [48]. We later estimated average effect size of nestedness (i.e. NODF index) of bacterial communities of hoopoes and of Z-values, and tested for possible influences of study area and treatments on the strength of the nestedness of communities. Statistical significance of average NODF values was inferred from the 95% CI of Z-values (i.e. whether or not it includes the threshold value of 1.64). The statistical GLM included the NODF values as dependent variable and geographic area, experimental treatment and the interaction as fixed effects. Since the performed experiment modified the bacterial community of nest materials, nestedness of communities including this microbiome were estimated only for control nests. Moreover, since microbiome of nests material of hoopoe nests was experimentally manipulated, we did not compare NODF values for pairs of bacterial communities that included nest material of experimental and control nests.

Finally, for multivariate analysis of communities directed to explore similarities among bacterial communities of hoopoe nests we did not consider rare OTUs (those that appeared in less than 4 samples in any of the bacterial communities considered) or nests with experimental materials. These analyses were performed by means of NPMANOVAs based of similarity matrices of Jaccard´s distance and 9999 permutations. Similarities among bacterial communities were shown in three dimensional figures from Principal Coordinates Analysis (PCoA) [50]. Both analyses were conducted by PAST Paleontological Statistics Software [51].

Study area did not explain a significant proportion of variance of any of the analyzed dependent factors (P > 0.1) and was therefore not considered in the final statistical models.

## Results

### Microbiomes of nest material, cloaca, uropygial gland, and eggshell of hoopoes

We identified 142 different OTUs (sizes between 100 bp and 819 bp); 101 of them were present in the uropygial gland, 58 in the cloaca, 91 in material of control nests, and 65 on the eggshell. On average, the number of OTUs detected differed among sampled sites (only control nests considered; F = 10.82, df = 3,44, p < 0.0001). The richest community was that of the uropygial secretion followed by those of the nest material, the eggshell, and the cloaca (Table 1).

**Table 1:** Average values of richness and nestedness microbiome. Average ± Standard Error (SE) of richness of microbiome of nest material (NM), uropygial secretion (US), cloaca (C) and eggshell (ES) of hoopoes. We also show average values (± SE) of degree of nestedness (NODF index) between pairs of microbiomes, excluding those with nest material (see Material and Methods). All these values were estimated for all studied nests (N = 24), and separately for nests with control (N = 12) and experimental (N = 12) materials. Results from statistical comparisons between control and experimental nests are also shown.

	All nests	Control	Experimental	F_1,22_	P
Microbiome Richness					
Nest material (NM)	9.46 (2.04)	16.50 (2.67)	2.42 (1.12)	23.72	0.001
Uropygial secretion (US)	22.17 (1.87)	22.83 (2.92)	21.50 (2.47)	0.12	0.730
Cloaca (C)	6.71 (1.10)	6.17 (1.60)	7.25 (1.55)	0.24	0.632
Eggshells (ES)	7.04 (4.67)	9.08 (1.64)	5.00 (0.60)	5.49	0.029
Microbiome Nestedness					
NODF [US(C)]	24.32 (5.81)	17.15 (6.47)	31.5 (9.50)	1.56	0.225
NODF [ES(US)]	28.23 (4.33)	30.14 (6.78)	26.32 (5.64)	0.19	0.669
NODF [ES(C)]	43.12 (8.15)	37.30 (10.45)	49.00(12.76)	0.50	0.491

Average ± Standard Error (SE) of richness of microbiome of nest material (NM), uropygial secretion (US), cloaca (C) and eggshell (ES) of hoopoes. We also show average values (±SE) of degree of nestedness (NODF index) between pairs of microbiomes, excluding those with nest material (see Material and Methods). All these values were estimated for all studied nests (N = 24), and separately for nests with control (N = 12) and experimental (N = 12) materials. Results from statistical comparisons between control and experimental nests are also shown.

**Fig 1:**
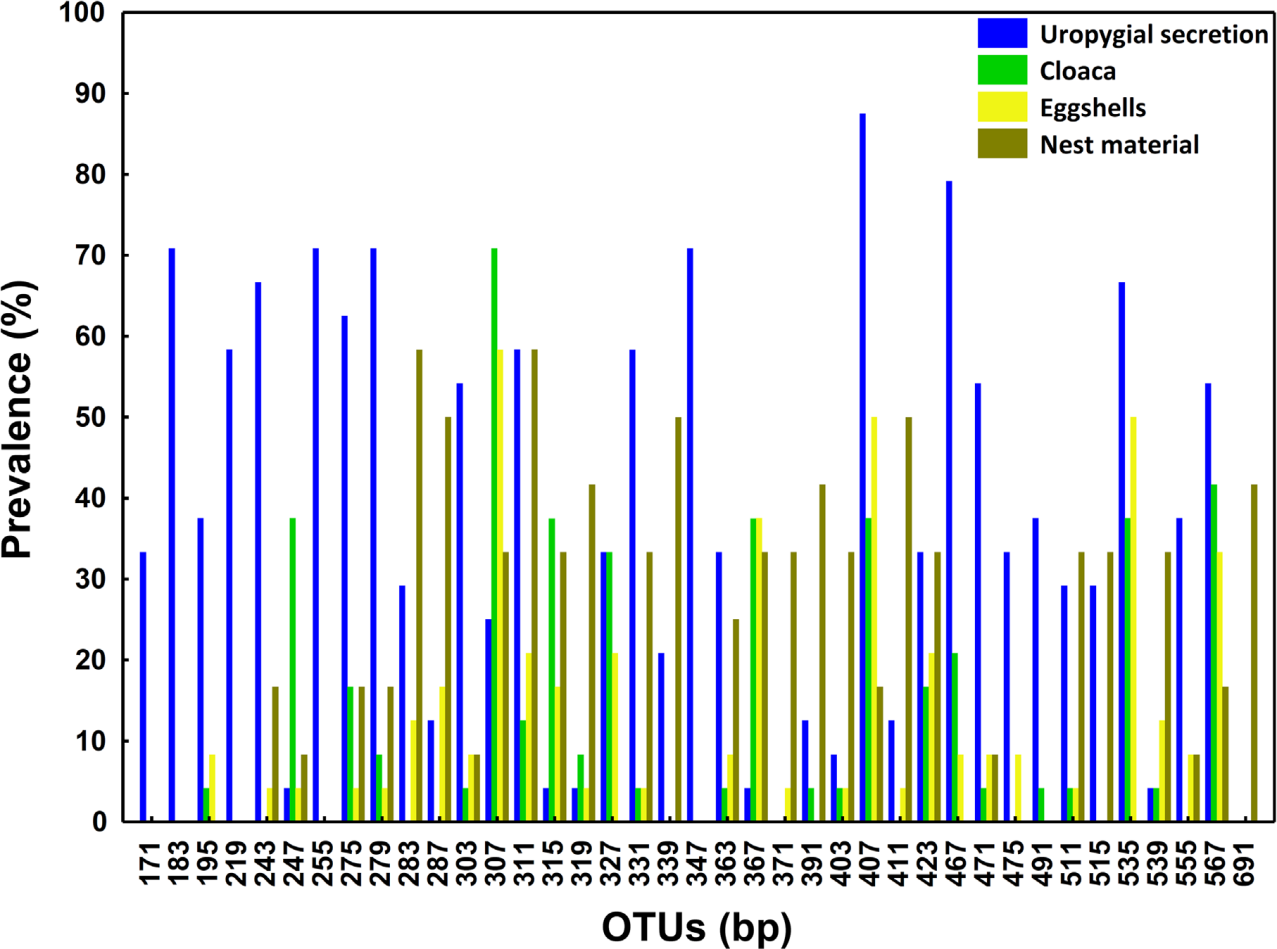
OTUs prevalence in bacterial samples from the uropygial gland, cloaca, eggshell and nest material. Prevalence (%) of different bacterial OTUs (named by their length in base pairs (bp)) found in more than 30% sampled uropygial glands (N = 24). We also show prevalence of these OTUs in the cloaca (N = 24), on the eggshells (N = 24) and in of the material of control nests (N = 12).

**Fig 2:**
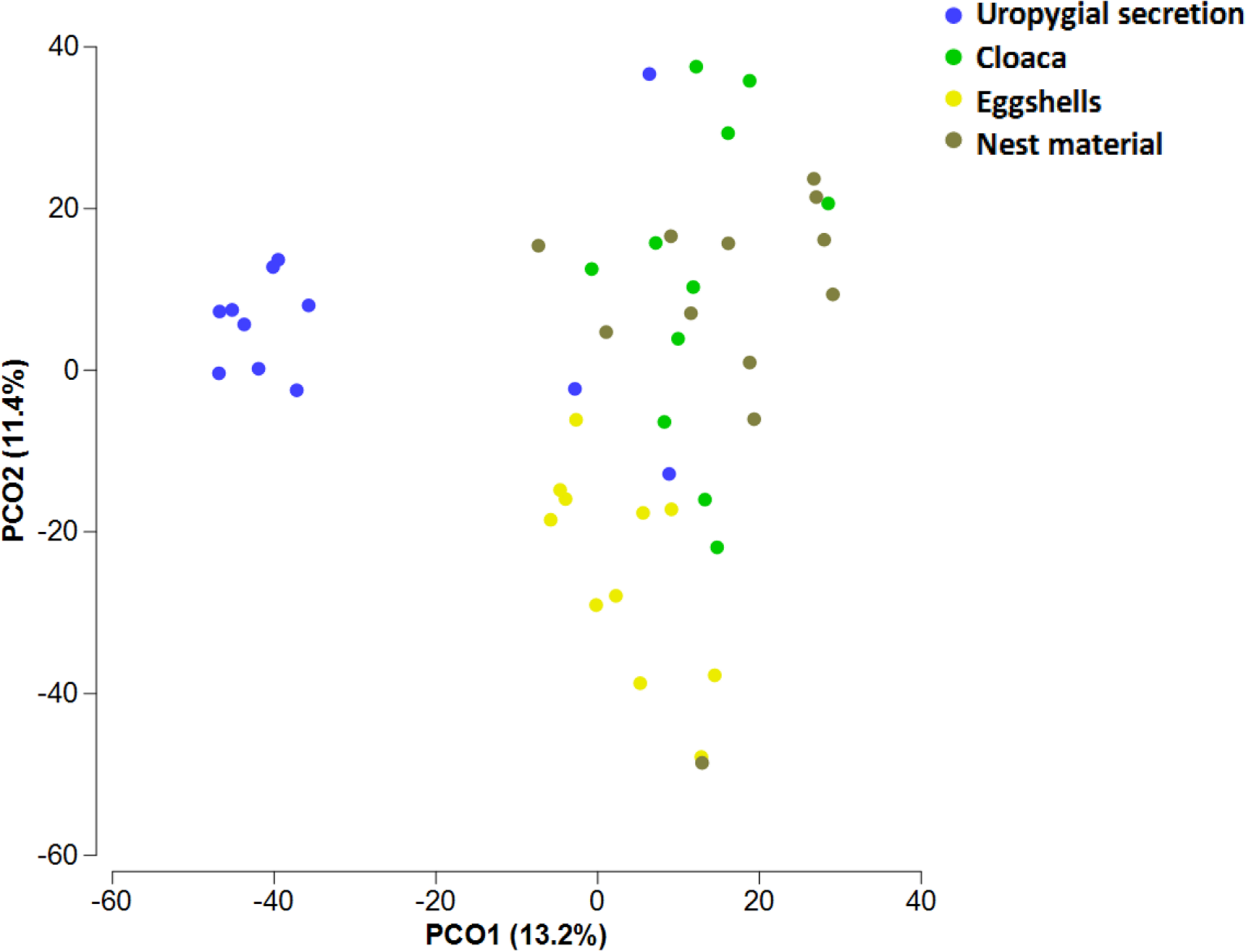
Similarities of bacterial communities in control nests. Multidimensional space representation (PCoA) based on similarities of communities harbored in female hoopoe uropygial gland, cloaca, eggshells and nest material of control nests. Variance captured by each axe is shown within the axis legends in parenthesis. The analysis was performed including only the OTUs present in uropygial secretion that were detected in at least 4 samples of any of the bacterial communities considered.

Of the 101 OTUs detected in uropygial glands, 45, 44 and 57, also occur in samples of the eggshells, cloaca, and materials of control nests, respectively. The detection of these OTUs in the bacterial communities of the uropygial gland did not depend of their presence in the nest materials or in the cloaca of the same nests (all χ^2^< 0.31, p > 0.25). When we took into consideration only the 39 OTUs present in more than 30% of some sampled sites, 5 OTUs were exclusive of uropygial secretion, 1 of nest material and no one was exclusive of cloaca or eggshells. OTU 307 appeared with highest prevalence in cloaca (70.83%) and on the eggshells (58.33%) (Fig 1), whereas OTUs 407 and 467 were the most commonly detected in samples of the uropygial secretion (89.5% and 79.17%, respectively) (Fig 1). For nest material microbiome, the most prevalent OTUs (> 50%) were the 283 and 311 (Fig 1). Bacterial communities of the uropygial gland, cloaca, eggshells, and nest material did separate significantly from each other in a multidimensional scale analysis (only control nests considered) (NPMANOVA, F = 3.81, df = 3,42, p < 0.001, Fig 2). Pairwise tests comparisons confirmed statistically significant differences among all sampled sites (t > 1.66, p < 0.002).

Finally, no evidence of nestedness of the microbiome of the uropygial secretion on that of the nest material (only control nests considered, all OTUs included, NODF(SE) = 15.01(8.51), Z(±CI) = -0.85–2.57, N = 12) or cloaca (Table 1, Z(±CI) = 0.78–4.04, N = 24) were detected, but the community of the eggshells was nested within the community of the secretion (Table 1, Z(±CI) = 2.08–8.47, N = 24). The eggshell bacterial community was not nested within those of the nest material (only control nests considered, all OTUs included, NODF(SE) = 15.51(4.62), Z(±CI) = -0.64–3.31, N = 12) or cloaca (Table 1, Z(±CI) = 0.18–3.26, N = 24). Finally, no evidence of nestedness among cloaca and nest material communities were detected (only control nests considered, all OTUs included, NODF(SE) = 9.28(4.10), (Z(±CI) = 0.77–4.52, N = 12). Consequently, no result supports the possibility that the microbiome of the nest material or that of the cloaca supplied the bacterial community of the uropygial secretion, but the uropygial secretion supplies the bacterial community of the eggshell of hoopoes.

### Experimental effects of nest material on nest microbiomes (uropygial secretion, cloaca and eggshells)

In accordance with the assumption of the experimental protocol, bacterial communities of nest material of experimental and control nests significantly differed in richness (Table 1) and composition (NPMANOVA, F = 2.34, df = 1,22, P = 0.007). However, we failed to detect the expected experimental effects on the microbiome of the uropygial secretion of nesting female hoopoes in terms of richness (Table 1) or composition (NPMANOVA, F = 0.61, df = 1,22, P = 0.895). Similarly, the experiment did not affect richness (Table 1) or composition (NPMANOVA, F = 1.97, df = 1,22, P = 0.510) of their cloaca microbiome.

**Fig 3:**
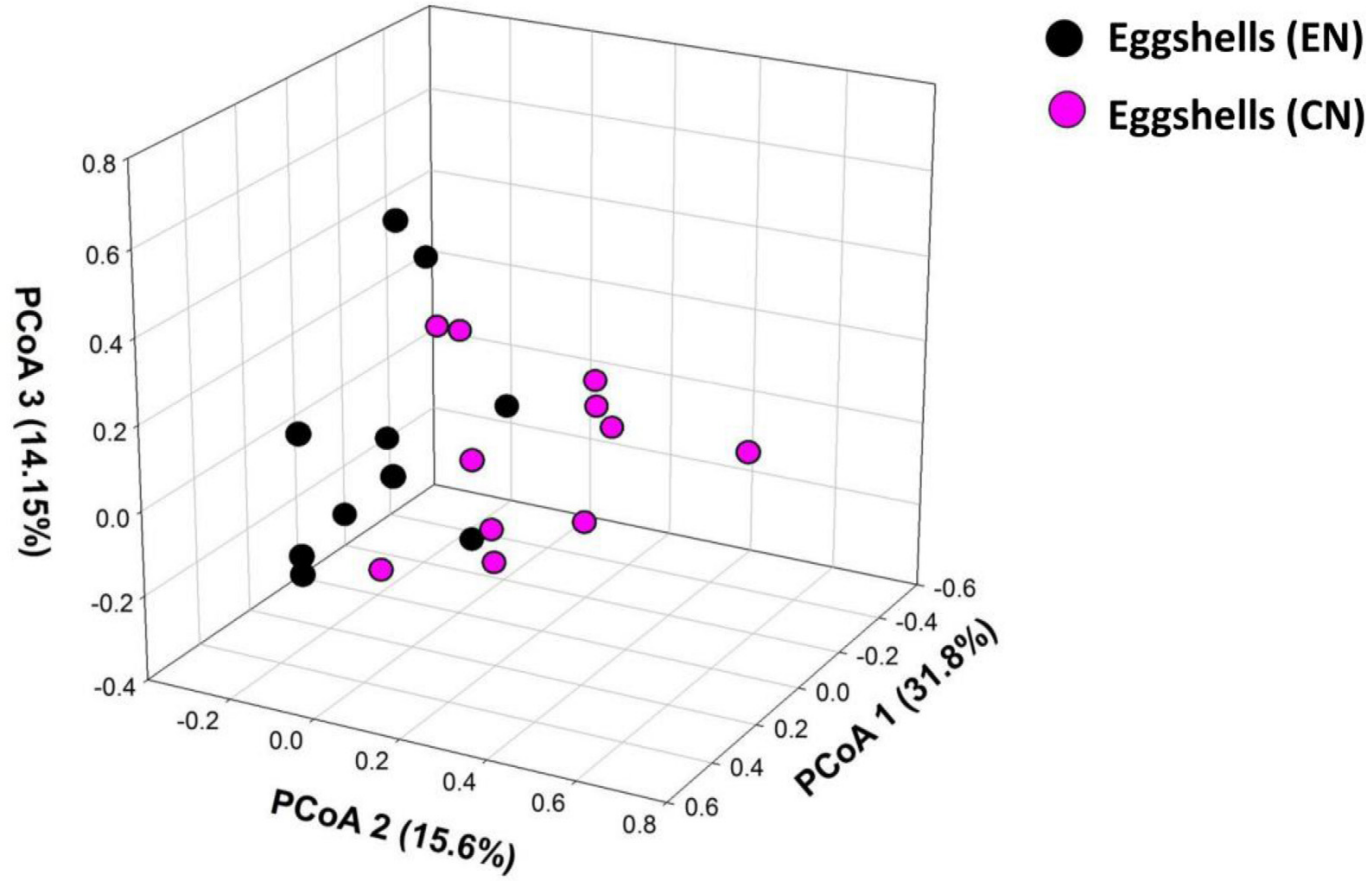
Similarity among bacterial communities of eggshells depending on type of nest material. Multidimensional space representation (PCoA) based on similarities of the composition of bacterial communities harbored on hoopoe eggshells in nests with control (CN) and experimental (EN) materials. Variance captured by each of the three axes is shown within the axis legends in parenthesis.

In agreement with the predicted effects of nest material on the microbiome of the eggshells, hoopoe eggshells in experimental nests harbored poorer bacterial communities (Table 1) that differed in composition from those of control nests (NPMANOVA, F = 3.63, df = 1,20, P = 0.005, Fig 3). Our experimental manipulation of nest material did not affect estimates of nestedness among these bacterial communities (Table 1).

## Discussion

We found consistent differences in the bacterial communities of different locations of hoopoes, eggs and nest materials. The bacterial community of the uropygial secretion of nesting females was the richest, and some of the bacterial strains detected there were also present in the nest material, on the eggshells, and in the female cloaca. However, the detection of one of these strains in the uropygial secretion did not depend on its presence in the nest material, or in the cloaca of the same female-nest, which suggest that neither, nest material or cloaca, are sources of bacterial strains for the communities of the uropygial gland of hoopoes. Finally, we found experimental evidence suggesting that the bacterial community of nest materials influence that of the eggshells of hoopoes. Below we discuss the importance of such results for the understanding of the associations among microbiomes of avian nests in general, and of those of hoopoes that particularly include the microbiome of their uropygial gland.

In a recently published work by Brandl et al. [5] they described complex bacterial assemblages within nests of reed warblers (*Acrocephalus scirpaceus*) with unique bacterial signatures detected for microbiomes of nest materials, cloaca, and eggshells. Similarly, we found statistically significant differences among microbial communities in hoopoe nests. These authors also showed correlative evidence suggesting that bacterial transmission across the nest components (i.e., from nest material to the eggshells) is likely to occur. Thus, different sampled locations (i.e., nest materials, cloaca, eggshells and uropygial secretion) should share some bacterial strains. Here, we also detected some strains that appeared in several nest locations which is accordance with the hypothesis that the microbiomes of different nest components are related to each other.

Hopooe females smear eggshells with uropygial secretion containing symbiotic bacteria that produce antimicrobial substances [24,25]. Thus, bacterial communities of the uropygial gland and eggshells of hoopoes should be related to each other. In accordance with this hypothesis we found evidence suggesting that the microbiome of the eggshell is nested within that of the uropygial oil (see also [26]). We have previously described significant associations in probability of detecting several OTUs (OTU307, OTU367, OTU407, OTU535, OTU567) on the eggshell and at different locations of the same females, including uropygial gland, beak and brood patch [54]. Interestingly, two of these OTUs (OTU307 and OTU367) were hardly detected in the uropygial secretion but quite frequently appeared in samples from the cloaca (see Results). Rather, the other three common OTUs on eggshells (i.e., OTU407, OTU535 and OTU567) were frequently detected in uropygial secretion samples. Consequently, these results may suggest that not only the microbiome of uropygial gland [26], but also that of the cloaca might be acting as sources of these bacteria for the eggshell. In accordance with this possibility in 75% of females presenting OTU307 on the eggshells (n = 16) this OTU was also detected in the cloaca.

However, mainly because the relatively low sample size, the association did not reach statistical significance (see Results). Further research is therefore necessary to confirm the hypothetical relationship among these particular microbial communities.

Eggshells are in contact with nest materials which would affect their microbiome, either because of antimicrobial activity of used materials acting on eggshells [9,10,55], or because some of the bacteria growing on nest material may colonize eggshells [5]. We did not find support for the last possibility since we did not detect the predicted association between presence of different OTUs on eggshells and in nest material samples of control nests. Rather, we detected experimental evidence supporting the hypothesis that nest materials influence the microbiome of the eggshells. We used commercial crushed olive stones as experimental nest material. This material demonstrated considerable antimicrobial properties against several indicator bacterial strains (Fig A in S2 Appendix) and, consequently, the antimicrobial activity of this material is the most likely explanation of the detected experimental effects on eggshell microbiome. Although it is commonly assumed in the literature that nest lining materials and nest sanitation behavior affect the bacterial environments where offspring develop (see Introduction), the influence of nest materials on eggshell bacterial community had never been tested experimentally. Eggshell bacterial load is commonly used as a proxy of probability of trans-shell embryo infection [52,53], and, therefore, our results are the first experimental evidence of nest materials influencing the microbiome experienced by avian embryos.

Nesting hoopoes harbor mutualistic bacteria in their uropygial gland [25,31] and, thus, exploring the association between microbiome of the uropygial gland and those from other nest locations may cast light on possible sources or reservoirs of mutualistic bacteria that colonize uropygial gland. Explaining how microbiomes are established and maintained in their animal hosts is a central question in biology, especially for those with beneficial effects [1,56]. In the case of hoopoes, symbiotic bacteria are mainly detected in uropygial gland secretion of nesting females and chicks [31] and, thus, bacterial acquisition apparently occurs each reproductive season and is likely related to the nest environment. Here, we tested the hypothesis that bacteria in the uropygial gland of incubating females were recruited from those in the nests material or in their cloaca, but found weak support for this possibility. We detected similarities among the microbial communities of nest materials, cloaca, eggshells and uropygial secretion of hoopoes that might suggest that bacteria from the cloaca and/or the nest material colonize the uropygial gland. However it is equally likely that these similarities were due to bacteria from the uropygial secretion of females reaching nest material and cloacal environment. In any case, we did not find statistical support for the expected associations; i.e., the detection of a particular bacterium in the uropygial secretion of females did not depend on its presence in nest materials or cloaca of the same females. Furthermore, the experimental manipulation of the microbial community of nest materials did not influence the microbiome of the uropygial secretion, which further supports that microbiomes of nest materials and of uropygial secretion are isolated from each other.

There are several possible explanations for the absence of evidence of inter-connections among the bacterial community of the uropygial secretion of hoopoes and those of nest materials and/or cloaca. We have previously found evidence of a genetic component explaining the enteroccoci community detected in the uropygial secretion of nestling hoopoes [57]. Thus, it is possible that stockpiles of symbiotic bacteria that come from their mothers were responsible for the bacteria in the uropygial secretion of reproducing females. This bacterial reservoir may be located in particular sites of the gut inaccessible for our cloacal samples. Another possible explanation is related to bacterial interference properties of different strains that are known to depend of environmental factors as resource availability, chemical environments, and bacterial community [56,58–60]. Hoopoes could collect bacteria from the nest material or from the gut and provide environments that select for bacterial strains with high antimicrobial capabilities (see [56]). It is therefore possible that strains that appeared at high density in the uropygial gland were at a very low density in nest material and/or or gut, making their detection in these last environments difficult by ARISA techniques [43]. Therefore further research is needed to robustly reject the hypothesis of a possible role of nest materials and cloaca as reservoir of symbiotic bacteria of the uropygial gland of hoopoes.

## Supporting Information

S1 Appendix Study of the bacterial load of the nest materials used in the experiment. Approximately the same amount of experimental (mean(SE) = 37.75(2.85) g) and control (mean(SE) = 37.75(2.85) g) material were diluted in 1mL of 0.2M pH7.2 phosphatase saline buffer. Bacterial load was estimated as number of Colony Forming Units (CFUs) in TSA media and serial dilutions. The estimates were adjusted to the volume of solution used for cultivation, and to the slight variation of weight of nest material employed for cultivation. Experimental material harbored bacteria at very low density (log10 transformed values, mean = 0.20,± 95%CI: -0.01,– 0.42, N = 10) in comparison with that of control material (log10 transformed values, mean = 5.10 ± 95% CI: 3.79,– 6.41, N = 10; F = 69.67, df = 1,18, P <0.001).
(DOCX)

S2 Appendix Study of the antimicrobial activity of the experimental material used (crushed olive stones). Antimicrobial activity of experimental nest material was tested against the following bacterial strains that included known pathogenic and keratinolytic bacteria: *Proteus sp*., *Escherichia coli*, *Mycobacterium sp*., *Bacillus licheniformis* D13, *Staphylococcus aureus*, *Klebsiella sp*., *Bacillus megatherium*, *Micrococcus luteus*, *Bacillus thuriguensis*, *Enterococcus faecalis* MRR-103, *Listeria monocytogenes* 4032, *Listeria inocua* CECT 340, *Lactobacillus plantarum* CECT 784, *Enterococcus faecium* 34, *Lactobacillus paracasei* 11–2, *Lactobacillus lactislactis* LM2301 (respectively strains 1, 3, 6, 7, 8, 9, 11, 12, 13, 16, 17, 18, 19, 21, 22, 23 Fig 1.1). Inhibitory activity of olive remains differed depending of the bacteria strains tested (Fig 1.2, F = 4.33, df = 15, 144, P <0.001), but was consistently higher than that of a control piece of crystal (Fig 1.2 F = 95.77, df = 2,144, P = 0.001). Average size of the inhibition halo (in mm) of sterilized and non-sterilized olive remains did not differ (suggesting that these properties are independent of the bacterial community associated (Fig 1.2). **Fig A Antimicrobial activity of nest material.** Average size of the antimicrobial activity (halo size in mm) of experimental nest material tested against pathogenic and keratinolytic bacteria (Bacteria tested: 1, 3, 6, 7, 8, 9, 11, 12, 13, 16, 17, 18, 19, 21, 22, 23). **Fig B. Antimicrobial activity of different nest material.** Average size of the antimicrobial activity (halo size in mm) of different material type (olive remains, sterilized and non-sterilized olive remains).
(DOCX)

## References

[1] McFall-Ngai M, Hadfield MG, Bosch TCG, Carey HV, Domazet-Lošo T, Douglas AE, et al Animals in a bacterial world, a new imperative for the life sciences. Proc Natl Acad Sci U S A. 2013; 110: 3229–3236. 10.1073/pnas.1218525110 23391737 PMC3587249

[2] Riley MA, Wertz JE. Bacteriocins: evolution, ecology, and application. Annu Rev Microbiol. 2002; 56: 117–137. 12142491 10.1146/annurev.micro.56.012302.161024

[3] Soler JJ, Martín-Vivaldi M, Peralta-Sánchez JM, Ruiz-Rodríguez M. Antibiotic-producing bacteria as a possible defence of birds against pathogenic microorganisms. Open Ornithol J. 2010; 3: 93–100.

[4] Clemente JC, Ursell LK, Parfrey LW, Knight R. The impact of the gut microbiota on human health: an integrative view. Cell. 2012; 148: 1258–1270. 10.1016/j.cell.2012.01.035 22424233 PMC5050011

[5] Brandl HB, van Dongen WFD, Darolová A, Krištofík J, Majtan J, Hoi H. Composition of bacterial assemblages in different components of reed warbler nests and a possible role of egg incubation in pathogen regulation. PLoS One. 2014; 9: e114861 10.1371/journal.pone.0114861 25493434 PMC4262450

[6] Bruce J, Drysdale EM. Trans-shell transmission In: Board R.G. and Fuller R. E, editors. Microbiology of avian eggs. London: Chapman & Hall; 1994 pp. 63–91.

[7] Barrow PA. The microflora of the alimentary tract and avian pathogens: translocation and vertical transmission In: Board R.G. and Fuller R, editors. In: Microbiology of avian eggs. London: Chapman & Hall; 1994 pp. 117–38.

[8] Shawkey MD, Pillai SR, Hill GE. Chemical warfare? Effects of uropygial oil on feather-degrading bacteria. J Avian Biol. 2003; 34: 345–349.

[9] Peralta-Sánchez JM, Møller AP, Martin-Platero AM, Soler JJ. Number and colour composition of nest lining feathers predict eggshell bacterial community in barn swallow nests: an experimental study. Funct Ecol. 2010; 24: 426–433.

[10] Ruiz-Castellano C, Tomás G, Ruiz-Rodríguez M, Martín-Gálvez D, Soler JJ. Nest Material Shapes Eggs Bacterial Environment. PLoS One. 2015; 11: e0148894. 10.1371/journal.pone.0148894 PMC4752222 26871451

[11] Peralta-Sánchez JM, Soler JJ, Martín-Platero AM, Knight R, Martínez-Bueno M, Møller AP. Eggshell bacterial load is related to antimicrobial properties of feathers lining barn swallow nests. Microb Ecol. 2014; 67: 480–487. 10.1007/s00248-013-0338-5 24317898

[12] Ibáñez-Álamo JD, Ruiz-Rodríguez M, Soler JJ. The mucous covering of fecal sacs prevents birds from infection with enteric bacteria. J Avian Biol. 2014; 45: 354–358.

[13] Ruiz-de-Castañeda R, Vela AI, Lobato E, Briones V, Moreno J. Bacterial Loads on Eggshells of the Pied Flycatcher: Environmental and Maternal Factors. Condor. 2011; 113: 200–208.

[14] Horrocks NP, Hine K, Hegemann A, Ndithia HK, Shobrak M, Ostrowski S, et al Are antimicrobial defences in bird eggs related to climatic conditions associated with risk of trans-shell microbial infection? Front Zool. 2014; 11: 49 10.1186/1742-9994-11-49 25057281 PMC4107615

[15] Peralta-Sánchez JM, Martín-Vivaldi M, Martín-Platero AM, Martínez-Bueno M, Oñate M, Ruiz-Rodríguez M, et al Avian life history traits influence eggshell bacterial loads: a comparative analysis. Ibis. 2012; 154: 725–737.

[16] Cook MI, Beissinger SR, Toranzos GA, Rodriguez RA, Arendt WJ. Microbial infection affects egg viability and incubation behavior in a tropical passerine. Behav Ecol. 2005; 16: 30–36.

[17] Shawkey MD, Firestone MK, Brodie EL, Beissinger SR. Avian incubation inhibits growth and diversification of bacterial assemblages on eggs. PLoS One. 2009; 4: e4522 10.1371/journal.pone.0004522 19225566 PMC2639702

[18] Lee WY, Kim M, Jablonski PG, Choe JC, Lee S. Effect of incubation on bacterial communities of eggshells in a temperate bird, the Eurasian Magpie (*Pica pica*). PLoS One. 2014; 9: e103959 10.1371/journal.pone.0103959 25089821 PMC4121233

[19] Clark L. The nest protection hypothesis: the adaptive use of plant secondary compounds by European starlings In: Loye J.E. and Zuk M, editors. In: Bird-parasite interactions: ecology, evolution and behaviour. Oxford: Oxford University Press; 1991 pp. 205–221.

[20] Mennerat A, Mirleau P, Blondel J, Perret P, Lambrechts MM, Heeb P. Aromatic plants in nests of the blue tit *Cyanistes caeruleus* protect chicks from bacteria. Oecologia. 2009; 161: 849–855. 10.1007/s00442-009-1418-6 19633988

[21] Martín-Platero AM, Valdivia E, Ruiz-Rodríguez M, Soler JJ, Martín-Vivaldi M, Maqueda M, et al Characterization of antimicrobial substances produced by *Enterococcus faecalis* MRR 10–3, isolated from the uropygial gland of the hoopoe (*Upupa epops*). Appl Environ Microbiol. 2006; 72: 4245–4249. 16751538 10.1128/AEM.02940-05 PMC1489579

[22] Martín-Vivaldi M, Peña A, Peralta-Sánchez JM, Sánchez L, Ananou S, Ruiz-Rodríguez M, et al Antimicrobial chemicals in hoopoe preen secretions are produced by symbiotic bacteria. Proc R Soc B. 2010; 277: 123–130. 10.1098/rspb.2009.1377 19812087 PMC2842625

[23] Ruiz-Rodríguez M, Martínez-Bueno M, Martín-Vivaldi M, Valdivia E, Soler JJ. Bacteriocins with a broader antimicrobial spectrum prevail in enterococcal symbionts isolated from the hoopoe’s uropygial gland. FEMS Microbiol Ecol. 2013; 85: 495–502. 10.1111/1574-6941.12138 23621827

[24] Soler JJ, Martín-Vivaldi M, Peralta-Sánchez JM, Arco L, Juárez-García-Pelayo N. Hoopoes color their eggs with antimicrobial uropygial secretions. Naturwissenschaften. 2014; 101: 697–705. 10.1007/s00114-014-1201-3 25011415

[25] Martín-Vivaldi M, Soler JJ, Peralta-Sánchez JM, Arco L, Martín-Platero AM, Martínez-Bueno M, et al Special structures of hoopoe eggshells enhance the adhesion of symbiont-carrying uropygial secretion that increase hatching success. J Anim Ecol. 2014; 83: 1289–1301. 10.1111/1365-2656.12243 24786478

[26] Soler JJ, Martínez-García Á, Rodríguez-Ruano S, Martínez-Bueno AM, Peralta-Sanchez JM, Martín-Vivaldi M. Nestedness of hoopoes’ bacterial communities: symbionts from the uropygial gland to the eggshell. Biol J Linn Soc. 2016; 10.1111/bij.12772

[27] Rehsteiner U. Abundance and habitat requirements of the Hoopoe *Upupa epops* in Extremadura (Spain). Ornithol Beobachter. 1996; 93: 277–287.

[28] Barbaro L, Couzi L, Bretagnolle V, Nezan J, Vetillard F. Multi-scale habitat selection and foraging ecology of the eurasian hoopoe (*Upupa epops*) in pine plantations. Biodivers Conserv. 2008; 17: 1073–1087.

[29] Schaub M, Martinez N, Tagmann-Ioset A, Weisshaupt N, Maurer ML, Reichlin TS, et al Patches of bare ground as a staple commodity for declining ground-foraging insectivorous farmland birds. PLoS One. 2010; 5: e13115 10.1371/journal.pone.0013115 20949083 PMC2950849

[30] Martín-Vivaldi M, Ruiz-Rodríguez M, Soler JJ, Peralta-Sánchez JM, Méndez M, Valdivia E, et al Seasonal, sexual and developmental differences in hoopoe *Upupa epops* preen gland morphology and secretions: evidence for a role of bacteria. J Avian Biol. 2009; 40: 191–205.

[31] Soler JJ, Martín-Vivaldi M, Ruiz-Rodríguez M, Valdivia E, Martín-Platero AM, Martínez-Bueno M, et al Symbiotic association between hoopoes and antibiotic-producing bacteria that live in their uropygial gland. Funct Ecol. 2008; 22: 864–871.

[32] Ruiz-Rodríguez M, Valdivia E, Martín-Vivaldi M, Martín-Platero AM, Martínez-Bueno M, Peralta-Sánchez JM, et al Antimicrobial activity and genetic profile of enteroccoci isolated from hoopoes uropygial gland. PLoS One. 2012; 7: e41843 10.1371/journal.pone.0041843 22911858 PMC3404078

[33] Martín-Vivaldi M, Palomino JJ, Soler M, Soler JJ. Determinants of reproductive success in the Hoopoe Upupa epops, a hole-nesting non-passerine bird with asynchronous hatching. Bird Study. 1999; 46: 205–216.

[34] Cramp S. The complete birds of the western Palearctic Optimedia, Oxford University Press, Oxford; 1998 Vol 4 p.

[35] Cruz-Peragón F, Palomar JM, Ortega A. Ciclo energético integral del sector oleícola en la provincia de Jaén (España). Grasas y aceites. 2006; 57: 219–228.

[36] Fleming HP, Walter WMJ, Etchells JL. Antimicrobial properties of oleuropein and products of its hydrolysis from green olives. Appl Microbiol. 1973; 26: 777–782. 4762397 10.1128/am.26.5.777-782.1973 PMC379901

[37] Aziz NH, Farag SE, Mousa LA, Abo-Zaid MA. Comparative antibacterial and antifungal effects of some phenolic compounds. Microbios. 1998; 93: 43–54. 9670554

[38] Ruiz-Rodríguez M, Valdivia E, Soler JJ, Martín-Vivaldi M, Martín-Platero AM, Martínez-Bueno M. Symbiotic bacteria living in the hoopoeʼs uropygial gland prevent feather degradation. J Exp Biol. 2009; 212: 3621–3626. 10.1242/jeb.031336 19880722

[39] Martín-Platero AM, Peralta-Sánchez JM, Soler JJ, Martínez-Bueno M. Chelex-based DNA isolation procedure for the identification of microbial communities of eggshell surfaces. Anal Biochem. 2010; 397: 253–255. 10.1016/j.ab.2009.10.041 19887062

[40] Fisher MM, Triplett EW. Automated approach for ribosomal intergenic spacer analysis of microbial diversity and its application to freshwater bacterial communities. Appl Environ Microbiol. 1999; 65: 4630–4636. 10508099 10.1128/aem.65.10.4630-4636.1999 PMC91617

[41] Danovaro R, Luna GM, Dell’Anno A, Pietrangeli B. Comparison of two fingerprinting techniques, terminal restriction fragment length polymorphism and automated ribosomal intergenic spacer analysis, for determination of bacterial diversity in aquatic environments. Appl Environ Microbiol. 2006; 72: 5982–5989. 16957219 10.1128/AEM.01361-06 PMC1563681

[42] Cardinale M, Brusetti L, Quatrini P, Borin S, Puglia AM, Rizzi A, et al Comparison of different primer sets for use in automated ribosomal intergenic spacer analysis of complex bacterial communities. Appl Environ Microbiol. 2004; 70: 6147–6156. 15466561 10.1128/AEM.70.10.6147-6156.2004 PMC522057

[43] Ramette A. Quantitative community fingerprinting methods for estimating the abundance of operational taxonomic units in natural microbial communities. Appl Environ Microbiol. 2009; 75: 2495–2505. 10.1128/AEM.02409-08 19201961 PMC2675222

[44] Bent SJ, Forney LJ. The tragedy of the uncommon: understanding limitations in the analysis of microbial diversity. ISME J. 2008; 2: 689–695. 10.1038/ismej.2008.44 18463690

[45] Loisel P, Harmand J, Zemb O, Latrille E, Lobry C, Delgenès J-P, et al Denaturing gradient electrophoresis (DGE) and single-strand conformation polymorphism (SSCP) molecular fingerprintings revisited by simulation and used as a tool to measure microbial diversity. Environ Microbiol. 2006; 8: 720–731. 16584483 10.1111/j.1462-2920.2005.00950.x

[46] Almeida-Neto M, Guimarães P, Guimarães PR Jr, Loyola RD, Ulrich W. A consistent metric for nestedness analysis in ecological systems: reconciling concept and measurement. Oikos. 2008; 117: 1227–1239.

[47] Almeida-Neto M, Ulrich W. A straightforward computational approach for measuring nestedness using quantitative matrices. Environ Model Softw. 2011; 26: 173–178.

[48] Strona G, Galli P, Seveso D, Montano S, Fattorini S. Nestedness for Dummies (NeD): A User-Friendly Web Interface for Exploratory Nestedness Analysis. J Stat Softw. 2014; 59: 1–9.26917999

[49] Traveset A, Kueffer C, Daehler CC. Global and regional nested patterns of non-native invasive floras on tropical islands. J Biogeogr. 2014; 41: 823–832.

[50] Legendre P, Legendre L. Numerical ecology. 2nd English. Amsterdam: Elsevier Science; 1998.

[51] Hammer Ø, Harper DAT, Ryan PD. PAST: Paleontological statistics software package for education and data analysis. Palaeontologia Electronica. 2001; 4: 9pp.

[52] Cook MI, Beissinger SR, Toranzos GA, Arendt WJ. Incubation reduces microbial growth on eggshells and the opportunity for trans-shell infection. Ecol Lett. 2005; 8: 532–537. 10.1111/j.1461-0248.2005.00748.x 21352457

[53] Soler JJ, Peralta-Sánchez JM, Martínez-Bueno M, Martín-Vivaldi M, Martín-Gálvez D, Vela AI, et al Brood parasitism is associated with increased bacterial contamination of host eggs: Bacterial loads of host and parasitic eggs. Biol J Linn Soc. 2011; 103: 836–848.

[54] Martínez-García Á; Soler JJ, Rodríguez-Ruano S, Martínez-Bueno M, Martín-Platero A, Juárez-García N, Martín-Vivaldi M. Microb. Ecol. Preening as a vehicle for key bacteria in hoopoes. 2015 10.1007/s00248-015-0636-1 26078039

[55] Møller A, Einar F-J, Willly M, Soler JJ. Host-parasite relationship between colonial terns and bacteria is modified by a mutualism with a plant with antibacterial defenses. Oecologia. 2013; 173: 169–178. 10.1007/s00442-013-2600-4 23404068

[56] Scheuring I, Yu DW. How to assemble a beneficial microbiome in three easy steps. Ecol Lett. 2012; 15: 1300–1307. 10.1111/j.1461-0248.2012.01853.x 22913725 PMC3507015

[57] Ruiz-Rodríguez M, Soler JJ, Martín-Vivaldi M, Martín-Platero AM, Méndez M, Peralta-Sánchez JM, et al Environmental factors shape the community of symbionts in the hoopoe uropygial gland more than genetic factors. Appl Environ Microbiol. 2014; 80: 6714–6723. 10.1128/AEM.02242-14 25172851 PMC4249055

[58] Poisot T, Lepennetier G, Martinez E, Ramsayer J, Hochberg ME. Resource availability affects the structure of a natural bacteria–bacteriophage community. Biol Lett. 2011; 7: 201–204. 10.1098/rsbl.2010.0774 20961886 PMC3061169

[59] Pérez-Gutiérrez R-A, López-Ramírez V, Islas Á, Alcaraz LD, Hernández-González I, Olivera BCL, et al Antagonism influences assembly of a Bacillus guild in a local community and is depicted as a food-chain network. ISME J. 2012; 7: 487–497. 10.1038/ismej.2012.119 23096405 PMC3578566

[60] Long RA, Rowley DC, Zamora E, Liu J, Bartlett DH, Azam F. Antagonistic interactions among marine bacteria impede the proliferation of *Vibrio cholerae*. Appl Environ Microbiol. 2005; 71: 8531–8536. 16332844 10.1128/AEM.71.12.8531-8536.2005 PMC1317384

