# Supplementary material for: Nest Bacterial Environment Affects Microbiome of Hoopoe Eggshells, but Not That of the Uropygial Secretion
Source: PLoS One. 2016 Jul 13;11(7):e0158158. doi: 10.1371/journal.pone.0158158 (PMC4943718; doi:10.1371/journal.pone.0158158)
Supplement: S1 Appendix — Approximately the same amount of experimental (mean(SE) = 37.75(2.85) g) and control (mean(SE) = 37.75(2.85) g) material were diluted in 1mL of 0.2M pH7.2 phosphatase saline buffer. Bacterial load was estimated as number of Colony Forming Units (CFUs) in TSA media and serial dilutions. The estimates were adjusted to the volume of solution used for cultivation, and to the slight variation of weight of nest material employed for cultivation. Experimental material harbored bacteria at very low density (log10 transformed values, mean = 0.20,± 95%CI: -0.01,– 0.42, N = 10) in comparison with that of control material (log10 transformed values, mean = 5.10 ± 95% CI: 3.79,– 6.41, N = 10; F = 69.67, df = 1,18, P <0.001). (DOCX) [file pone.0158158.s001.docx]

**S1 Appendix. Study of the bacterial load of the nest materials used in the experiment.** Approximately the same amount of experimental (mean(SE) = 37.75(2.85) g) and control (mean(SE) = 37.75(2.85) g) material were diluted in 1mL of 0.2M pH7.2 phosphatase saline buffer. Bacterial load was estimated as number of Colony Forming Units (CFUs) in TSA media and serial dilutions. The estimates were adjusted to the volume of solution used for cultivation and to the slight variation of weight of nest material employed for cultivation. Experimental material harbored bacteria at very low density (log10 transformed values, mean = 0.20,± 95%CI: -0.01, – 0.42, N = 10) in comparison with that of control material (log10 transformed values, mean = 5.10 ± 95% CI: 3.79, – 6.41, N = 10; F = 69.67, df = 1,18, P <0.001).
