# Supplementary material for: Nest Bacterial Environment Affects Microbiome of Hoopoe Eggshells, but Not That of the Uropygial Secretion
Source: PLoS One. 2016 Jul 13;11(7):e0158158. doi: 10.1371/journal.pone.0158158 (PMC4943718; doi:10.1371/journal.pone.0158158)

**S2 Appendix. Study of the antimicrobial activity of the experimental material used (crushed olive stones).** Antimicrobial activity of experimental nest material was tested against the following bacterial strains that included known pathogenic and keratinolytic bacteria: *Proteus sp.*, *Escherichia coli*, *Mycobacterium sp.*, *Bacillus licheniformis* D13, *Staphylococcus aureus*, *Klebsiella sp.*, *Bacillus megatherium*, *Micrococcus luteus*, *Bacillus thuriguensis*, *Enterococcus faecalis* MRR-103, *Listeria monocytogenes* 4032, *Listeria inocua* CECT 340, *Lactobacillus plantarum* CECT 784, *Enterococcus faecium* 34, *Lactobacillus paracasei* 11-2, *Lactobacillus lactis lactis* LM2301 (respectively strains 1, 3, 6, 7, 8, 9, 11, 12, 13, 16, 17, 18, 19, 21, 22, 23 Fig. 1.1). Inhibitory activity of olive remains differed depending of the bacteria strains tested (Fig. 1.2, F = 4.33, df = 15, 144, P <0.001), but was consistently higher than that of a control piece of crystal (Fig. 1.2 F = 95.77, df = 2,144, P = 0.001). Average size of the inhibition halo (in mm) of sterilized and non-sterilized olive remains did not differ (suggesting that these properties are independent of the bacterial community associated (Fig. 1.2).

**Figure A. Antimicrobial activity of nest material.** Average size of the antimicrobial activity (halo size in mm) of experimental nest material tested against pathogenic and keratinolytic bacteria (Bacteria tested: 1, 3, 6, 7, 8, 9, 11, 12, 13, 16, 17, 18, 19, 21, 22, 23)


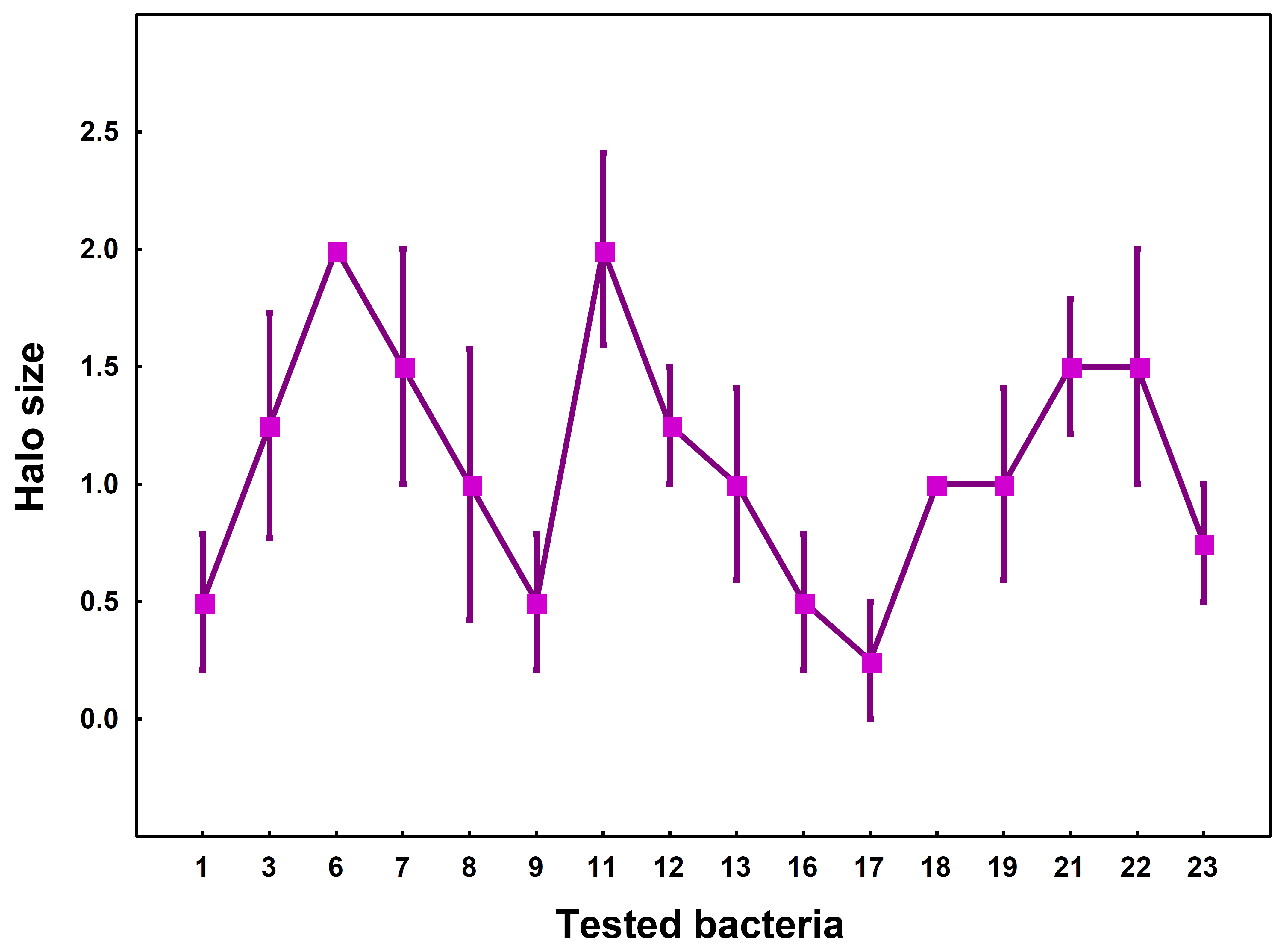


**Figure B. Antimicrobial activity of different nest material.** Average size of the antimicrobial activity (halo size in mm) of different material type (olive remains, sterilized and non-sterilized olive remains).


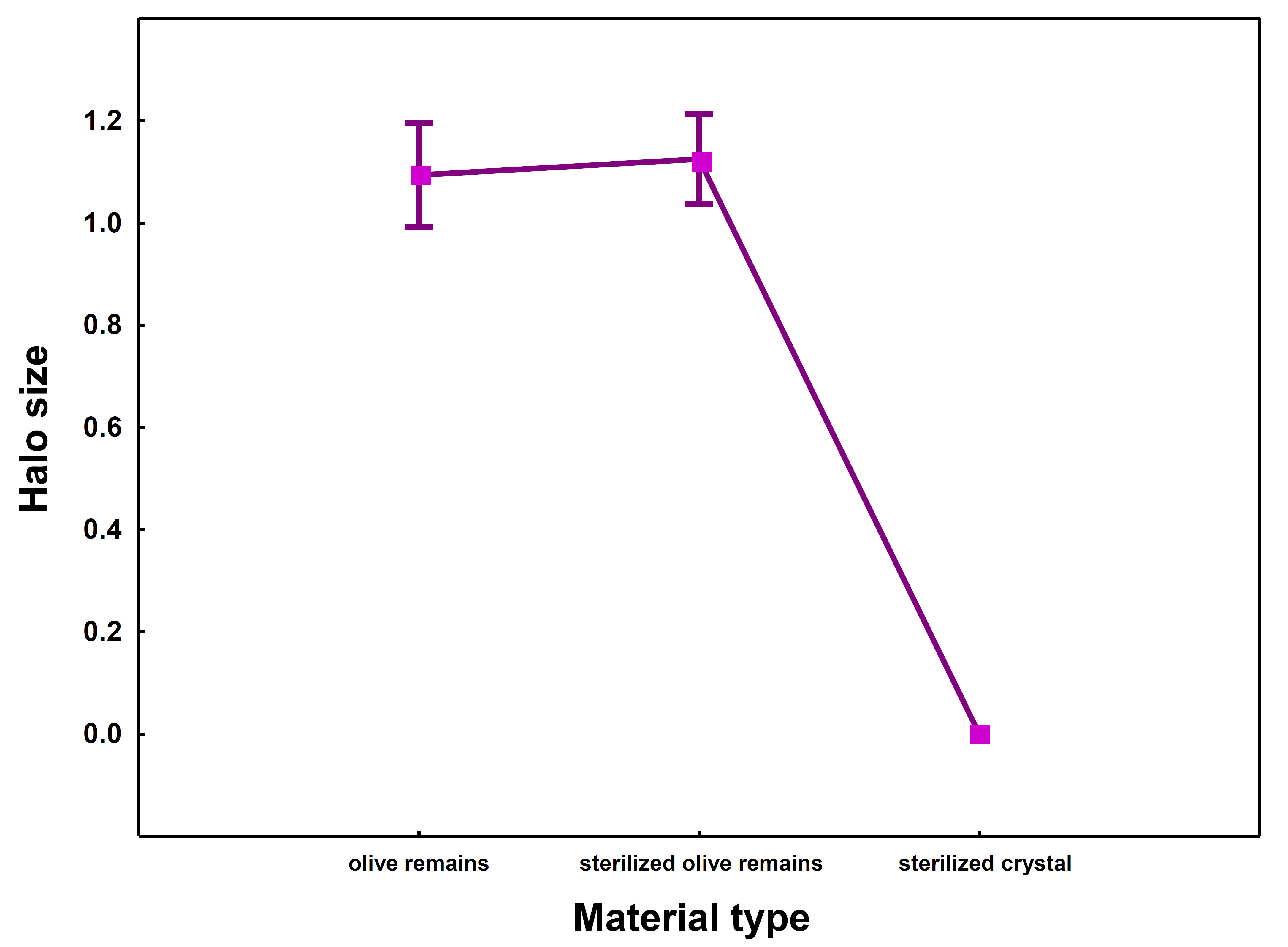

Supplement: S2 Appendix — Antimicrobial activity of experimental nest material was tested against the following bacterial strains that included known pathogenic and keratinolytic bacteria: Proteus sp., Escherichia coli, Mycobacterium sp., Bacillus licheniformis D13, Staphylococcus aureus, Klebsiella sp., Bacillus megatherium, Micrococcus luteus, Bacillus thuriguensis, Enterococcus faecalis MRR-103, Listeria monocytogenes 4032, Listeria inocua CECT 340, Lactobacillus plantarum CECT 784, Enterococcus faecium 34, Lactobacillus paracasei 11–2, Lactobacillus lactislactis LM2301 (respectively strains 1, 3, 6, 7, 8, 9, 11, 12, 13, 16, 17, 18, 19, 21, 22, 23 Fig 1.1). Inhibitory activity of olive remains differed depending of the bacteria strains tested (Fig 1.2, F = 4.33, df = 15, 144, P <0.001), but was consistently higher than that of a control piece of crystal (Fig 1.2 F = 95.77, df = 2,144, P = 0.001). Average size of the inhibition halo (in mm) of sterilized and non-sterilized olive remains did not differ (suggesting that these properties are independent of the bacterial community associated (Fig 1.2). Fig A Antimicrobial activity of nest material. Average size of the antimicrobial activity (halo size in mm) of experimental nest material tested against pathogenic and keratinolytic bacteria (Bacteria tested: 1, 3, 6, 7, 8, 9, 11, 12, 13, 16, 17, 18, 19, 21, 22, 23). Fig B. Antimicrobial activity of different nest material. Average size of the antimicrobial activity (halo size in mm) of different material type (olive remains, sterilized and non-sterilized olive remains). (DOCX) [file pone.0158158.s002.docx]
